# Routine Vaccination Coverage — Worldwide, 2021

**DOI:** 10.15585/mmwr.mm7144a2

**Published:** 2022-11-04

**Authors:** Audrey Rachlin, M. Carolina Danovaro-Holliday, Padraic Murphy, Samir V. Sodha, Aaron S. Wallace

**Affiliations:** ^1^Epidemic Intelligence Service, CDC; ^2^Global Immunization Division, Center for Global Health, CDC; ^3^Department of Immunization, Vaccines and Biologicals, World Health Organization, Geneva, Switzerland; ^4^Division of Data, Analytics, Planning and Monitoring, UNICEF, New York, New York.

In 2020, the World Health Assembly endorsed the Immunization Agenda 2030, an ambitious global immunization strategy to reduce morbidity and mortality from vaccine-preventable diseases (*1*). This report updates a 2020 report (*2*) with global, regional,* and national vaccination coverage estimates and trends through 2021. Global estimates of coverage with 3 doses of diphtheria-tetanus-pertussis–containing vaccine (DTPcv3) decreased from an average of 86% during 2015–2019 to 83% in 2020 and 81% in 2021. Worldwide in 2021, 25.0 million infants (19% of the target population) were not vaccinated with DTPcv3, 2.1 million more than in 2020 and 5.9 million more than in 2019. In 2021, the number of infants who did not receive any DTPcv dose by age 12 months (18.2 million) was 37% higher than in 2019 (13.3 million). Coverage with the first dose of measles-containing vaccine (MCV1) decreased from an average of 85% during 2015–2019 to 84% in 2020 and 81% in 2021. These are the lowest coverage levels for DTPcv3 and MCV1 since 2008. ​Global coverage estimates were also lower in 2021 than in 2020 and 2019 for bacillus Calmette-Guérin vaccine (BCG) as well as for the completed series of *Haemophilus influenzae* type b vaccine (Hib), hepatitis B vaccine (HepB), polio vaccine (Pol), and rubella-containing vaccine (RCV). The COVID-19 pandemic has resulted in disruptions to routine immunization services worldwide. Full recovery to immunization programs will require context-specific strategies to address immunization gaps by catching up missed children, prioritizing essential health services, and strengthening immunization programs to prevent outbreaks (*3*).

The World Health Organization (WHO) established the Expanded Programme on Immunization in 1974 to protect infants against six diseases through vaccination (e.g., BCG, DTP, Pol, and MCV) (*4*). Since then, additional vaccines and vaccine doses have been introduced during the first year of life (e.g., HepB, Hib, pneumococcal conjugate vaccine [PCV], RCV, and rotavirus) and at older ages (e.g., human papillomavirus [HPV] vaccine in females) (*4*). WHO and UNICEF produce annual estimates of immunization coverage through review of available country-specific data, including administrative and survey-based coverage^†^^,^^§^ (*5*). DTPcv3 coverage by age 12 months is an indicator of routine immunization program performance, and DTPcv3, MCV2, 3 doses of PCV (PCV3), and HPV vaccine are indicators for the Sustainable Development Goals.^¶^ Children who have not received any doses of DTPcv by age 12 months (zero-dose children) represent those with poor access to immunization and other essential health services. Children who receive the first DTPcv dose (DTPcv1) but do not complete the full series are considered incompletely vaccinated. 

WHO and UNICEF global estimates of national immunization coverage for DTPcv1 decreased from 90% in 2019 to 87% in 2020 and 86% in 2021, the lowest level since 2005. In 2021, DTPcv1 coverage ranged from 80% in the WHO African Region to 97% in the European Region (Table 1). DTPcv3 coverage followed similar regional trends. The decline in first and third dose DTPcv coverage during 2019–2021 was largest in the South-East Asia Region (from 94% to 86% for DTPcv1 and from 91% to 82% for DTPcv3). In the Americas, DTPcv1 and DTPcv3 coverage decreased by 3 and 4 percentage points, respectively, during 2019–2021 (Figure). Among the 194 WHO member states, DTPcv1 coverage during 2019–2021 was stable or declined in 170 (88%); DTPcv3 coverage during this period was stable or declined in 167 member states (86%).

**TABLE 1:** Estimated vaccination coverage, by World Health Organization region and vaccine — worldwide, 2021

Vaccine	Countries with vaccine in schedule,* no. (%)	WHO region coverage,^†,§,¶^ %						
		Global	African	Americas	Eastern Mediterranean	European	South-East Asia	Western Pacific
BCG	156 (80)	84	78	81	88	92	85	89
DTPcv1	194 (100)	86	80	86	89	97	86	91
DTPcv3	194 (100)	81	71	80	82	94	82	90
HepB BD	111 (57)	42	17	59	33	43	51	78
HepB3	190 (98)	80	71	80	82	91	82	90
Hib3	192 (99)	71	71	79	82	81	82	29
HPV, last**	116 (60)	12	21	38	—	27	2	2
MCV1	194 (100)	81	68	84	82	94	86	91
MCV2	183 (94)	71	41	75	77	91	78	91
PCV3	154 (79)	51	66	74	54	82	29	19
Pol3	194 (100)	80	70	79	83	94	82	90
RCV1	173 (89)	66	35	84	42	94	86	91
Rota, last^††^	118 (61)	49	52	69	57	34	61	2

**Abbreviations:** BCG = Bacille Calmette-Guérin vaccine; DTPcv1 = first dose of diphtheria-tetanus-pertussis–containing vaccine; DTPcv3 = third dose of diphtheria-tetanus-pertussis vaccine; HepB BD = birth dose of hepatitis B vaccine; HepB3 = third dose of hepatitis B vaccine; Hib3 = third dose of *Haemophilus influenzae* type b vaccine; HPV, last = final dose of human papillomavirus vaccine; MCV1 = first dose of measles-containing vaccine; MCV2 = second dose of MCV; PCV3 = third dose of pneumococcal conjugate vaccine; Pol3 = third dose of polio vaccine; RCV1 = first dose of rubella-containing vaccine; Rota, last = final dose of rotavirus vaccine series; WHO = World Health Organization.

* Does not include countries recommending vaccines for special groups only.

^†^ Based on WHO regional classifications. https://www.who.int/about/who-we-are/regional-offices

^§^ https://www.who.int/teams/immunization-vaccines-and-biologicals/policies/who-recommendations-for-routine-immunization---summary-tables

^¶^ Included countries are 194 WHO member states. BCG coverage is based on 156 countries with BCG in the national schedule, whereas coverage for all other vaccines is based on 194 countries (global) or all countries in the specified region. Administrative coverage is the number of vaccine doses administered to those in a specified target group divided by the estimated target population. During vaccination coverage surveys, a representative sample of households are visited and caregivers of children in a specified target group (e.g., aged 12–23 months) are interviewed. Dates of vaccination are transcribed from the child's home-based record, from health facility records, or recorded based on caregiver recall. Survey-based vaccination coverage is calculated as the proportion of persons in a target age group who received a vaccine dose.

** Estimates based on last dose given among females. Number of doses to complete the HPV series depends on age of recipient.

^††^ Number of doses to complete the rotavirus vaccine series varies between 2 and 3 depending on vaccine products.

**FIGURE:**
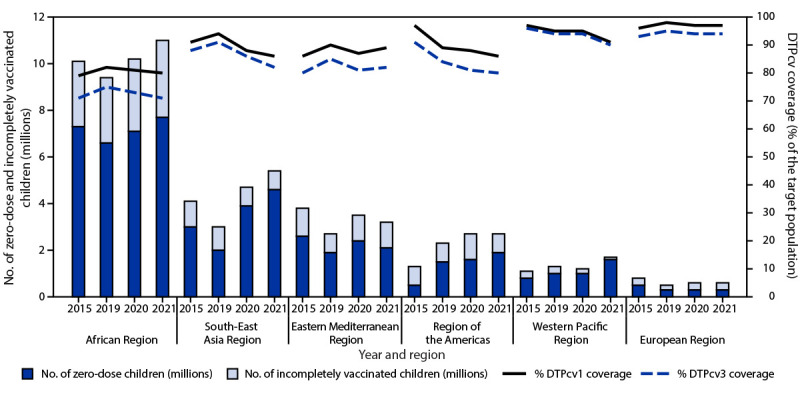
Estimated number of zero-dose and incompletely vaccinated children* and estimated coverage with first and third dose of diphtheria-tetanus-pertussis–containing vaccine, by World Health Organization region — worldwide, 2015 and 2019–2021 **Abbreviations:** DTPcv1 = first dose of diphtheria-tetanus-pertussis–containing vaccine; DTPcv3 = third dose of diphtheria-tetanus-pertussis–containing vaccine. * Zero-dose children are surviving infants who lack receipt of any dose of DTPcv by age 12 months. Incompletely vaccinated children are those who received at least 1 dose, but not the third dose needed for basic protection.

In 2021, 25.0 million children worldwide had not completed the 3-dose DTPcv series, 2.1 million more than in 2020 (22.9 million) and 5.9 million more than in 2019 (19.1 million); 18.2 million (73%) had received no doses, and 6.8 million (27%) were incompletely vaccinated with DTPcv. The number of zero-dose children was unevenly distributed by WHO region, economic classification,** and country eligibility for support from Gavi, the Vaccine Alliance (Gavi)^††^ (Table 2) (Figure). Among 18.2 million zero-dose children in 2021, low-income countries accounted for 5.0 million (27%), whereas middle-income countries had the largest number (12.8 million; 70%). Ten countries (43% of the global birth cohort) accounted for 62% (11.4 million) of zero-dose children: India (2.7 million), Nigeria (2.2 million), Indonesia (1.1 million), Ethiopia (1.1 million), Philippines (1.0 million), Democratic Republic of the Congo (0.73 million), Brazil (0.71 million), Pakistan (0.61 million), Angola (0.55 million), and Burma (0.49 million). Approximately 12 million zero-dose children (69% of the global total) lived in Gavi-eligible countries. DTPcv3 coverage declined sharply in 17 countries that transitioned out of Gavi support,^§§^ from a weighted average of 82% in 2019 to 70% in 2021, whereas those supported by Gavi were less affected (weighted average of 82% in 2019 compared with 77% in 2021).

**TABLE 2:** Number and global percentage of zero-dose children,* by World Health Organization region; World Bank economic classification; and Gavi, the Vaccine Alliance eligibility — worldwide, 2015 and 2019–2021

Characteristic, yr	WHO region^†^							Economic classification^¶^			Among Gavi-eligible countries^§^
	Global	Africa	Americas	Eastern Mediterranean	European	South-East Asia	Western Pacific	Low	Middle	High	
**2015**											
No. of countries	194	47	35	21	53	11	27	31	104	57	57
No. of surviving infants (millions)	138.5	34.7	14.8	18.4	11.5	34.7	24.4	21.5	103.6	12.9	72.8
Global % of surviving infants	—	25	11	13	8	25	18	16	75	9	53
No. of zero-dose children (millions)	14.7	7.3	0.5	2.6	0.5	3.0	0.8	3.9	10.4	0.3	11.7
Global % of zero-dose children	—	50	3	17	3	21	6	27	71	2	80
**2019**											
No. of countries	194	47	35	21	53	11	27	29	103	60	57
No. of surviving infants (millions)	134.3	37.0	14.0	18.1	10.5	33.3	21.4	23.1	98.9	12.0	74.3
Global % of surviving infants	—	28	10	14	8	25	16	17	74	9	55
No. of zero-dose children (millions)	13.3	6.6	1.5	1.9	0.3	2.0	1.1	3.9	9.0	0.3	9.3
Global % of zero-dose children	—	50	12	14	2	15	8	29	68	2	70
**2020**											
No. of countries	194	47	35	21	53	11	27	27	108	57	57
No. of surviving infants (millions)	131.6	37.5	13.7	18.2	10.3	32.8	19.0	23.6	95.7	11.8	74.6
Global % of surviving infants	—	29	10	14	8	25	15	18	73	9	57
No. of zero-dose children (millions)	16.5	7.1	1.6	2.4	0.3	3.9	1.0	4.3	11.8	0.3	11.9
Global % of zero-dose children	—	43	10	15	2	24	6	26	72	2	72
**2021**											
No. of countries	194	47	35	22	53	11	27	28	106	58	57
No. of surviving infants (millions)	130.5	38.1	13.6	18.2	10.2	32.8	17.6	24.0	94.2	11.8	75.2
Global % of surviving infants	—	29	10	14	8	25	13	18	72	9	58
No. of zero-dose children (millions)	18.2	7.7	1.9	2.1	0.3	4.6	1.6	5.0	12.8	0.3	12.5
Global % of zero-dose children	—	42	10	11	2	25	9	27	70	2	69

**Abbreviations:** DTPcv3 = third dose of diphtheria-tetanus-pertussis–containing vaccine; Gavi = Gavi, the Vaccine Alliance; GNI = gross national income; WHO = World Health Organization.

* Zero-dose children are surviving infants who lack receipt of any dose of DTPcv by age 12 months. The 2021 WHO and UNICEF estimates of national immunization coverage used the 2022 revision of the World Population Prospect from the United Nations Population Division for estimates of national immunization coverage and for calculations of regional and global vaccination coverage figures. Estimates of live births and surviving infants changed for previous years. The changes in target population estimates result in a 1%-point lower global DTPcv3 coverage than if calculations had used data from the 2019 revision and 2%-point lower regional average DTPcv3 coverage for the World Population Prospect.

^†^ Included countries are WHO member states.

^¶^ GNI per capita is calculated using the World Bank Atlas method in U.S. dollars. For all years shown, Cook Islands and Niue are not included because of lack of available GNI estimates. For 2020 and 2021, data for Venezuela were also temporarily unclassified pending release of revised national accounts statistics. https://datahelpdesk.worldbank.org/knowledgebase/articles/906519-world-bank-country-and-lending-groups

^§^ Gavi is a public-private global health partnership that aims to increase access to immunization in poor countries. Based on Gavi 5.0 (2021–2025), eligibility includes 57 low- and middle-income countries eligible to receive financial assistance through grants contingent on a country’s GNI per capita. Eligibility is defined as a country’s average 3-year GNI per capita in ≤1,630 U.S. dollars. As GNI increases, a country moves through Gavi’s different eligibility phases until reaching the transition phase in which GNI exceeds the eligibility threshold. https://www.gavi.org

Global MCV1 coverage remained stable during 2015–2019 (85%–86%) but decreased to 83% in 2020 and to 81% in 2021. The largest declines in MCV1 coverage during 2019–2021 occurred in the South-East Asia Region (from 94% to 86%) and the Western Pacific Region (from 95% to 91%) (Table 1). During 2015–2019, coverage with 2 MCV doses (MCV2) increased from 63% to 71%, reflecting second dose introductions in many countries.^¶¶^ However, MCV2 coverage remained stable thereafter (72% in 2020 and 71% in 2021), with only four additional countries introducing MCV2 during 2020–2021.

Global coverage during 2019–2021 decreased for all of the following recommended childhood vaccines: BCG, from 88% to 84%; the completed Hib series, from 73% to 71%; RCV, from 69% to 66%; 3-dose HepB series, from 85% to 80%; HepB birth dose, from 44% to 42%; and the third Pol dose, from 86% to 80%. Global coverage with first dose of HPV vaccine among females declined from 20% in 2019 to 15% in 2021, and with the last dose, from 14% in 2019 to 12% in 2021. Global PCV3 coverage was stagnant (50% in 2019, 51% in 2020, and 51% in 2021), and coverage with the final dose of rotavirus vaccine series increased from 40% in 2019 to 49% in 2021.***

## Discussion

 Since the start of the COVID-19 pandemic in 2020, a widespread decline in childhood vaccinations has occurred globally, putting millions of additional children at risk for vaccine-preventable diseases. Global DTPcv3 coverage declined by 5 percentage points during 2019–2021, meaning that at least 22.9 million children in 2020 and 25.0 million children in 2021 did not access or fully utilize routine immunization services. Immunization outreach services were particularly affected (*6*), and the most vulnerable populations have experienced the largest impact. Among all WHO regions, the largest declines in DTPcv3 and MCV1 coverage occurred in the South-East Asia Region.

The continued decline in vaccination coverage during 2020–2021 was likely a result of many factors, including strained health systems caused by the COVID-19 pandemic, coupled with delivery of COVID-19 vaccines. These stresses have led to challenges with supply chains, human resources, and financing. Increasing vaccine misinformation, disinformation, and hesitancy also likely contributed to declines in some countries (*6*). The risk of vaccine-preventable disease outbreaks is likely to persist if urgent action is not taken to recover immunization program losses.

Expanding immunization services to reach zero-dose and incompletely vaccinated children and reducing immunization inequities are key objectives of the Immunization Agenda 2030 (*1*). Gavi has provided support for vaccines and vaccination services to low- and lower-middle income countries since 2000, helping to improve access and reduce disparities in immunization coverage with high-income countries (*7*). However, during 2019–2021, vaccination coverage declined more sharply in countries that transitioned out of Gavi support than in those supported by Gavi, underscoring the vulnerability of these systems. As countries develop economically, they potentially become less eligible for external funding and require increased domestic financing for immunization. In times of crisis, such as during the COVID-19 pandemic, middle-income countries, which account for an increasing share of unprotected children, might be unable to allocate sufficient resources to immunization programs to reach every child with available vaccines.

The findings in this report are subject to at least five limitations. First, for 18 countries (6% of the global birth cohort) that did not report immunization coverage data for 2021 by July 7, 2022, estimated coverage for 2020 was used.^†††^ Second, because COVID-19 also disrupted survey implementation, estimates for 2021 are less determined by survey data than are estimates for previous years. Third, the estimated numbers of zero-dose and incompletely vaccinated children rely on population estimates that are subject to inaccuracies. Fourth, data quality limitations in some countries might have resulted in inaccurate estimates of administrative coverage, and selection and recall bias could affect survey-based estimates of coverage (*5*). Finally, coverage estimates do not include statistical uncertainty.

Reversing worrisome vaccination trends and extending previous gains in coverage beyond prepandemic levels will require targeted and context-specific approaches to eliminate barriers to vaccination, particularly in communities with large populations of zero-dose children. WHO has published strategies and guiding principles for implementing catch-up vaccination and recovering essential immunization services (*8*–*10*). Countries are urged to recover immunization services while capitalizing on opportunities from the pandemic response and COVID-19 vaccine rollout to strengthen routine immunization services and increase primary health care resiliency. This can be achieved by prioritizing routine immunization as an essential health service, improving access to vaccination across the life span, strengthening data systems, safeguarding sustainable immunization financing, and building vaccine confidence.

**Summary**

**What is already known about this topic?**

High routine childhood vaccination coverage achieved during 2015–2019 declined globally for most vaccines during 2019–2021 because of COVID-19 pandemic disruptions.

**What is added by this report?**

In 2021, the estimated global coverage with 3 doses of diphtheria-tetanus-pertussis–containing vaccine as well as the first dose of measles-containing vaccine decreased to 81%, the lowest level since 2008. Globally, 25.0 million children were unvaccinated or incompletely vaccinated in 2021, 5.9 million more than in 2019.

**What are the implications for public health practice?**

Reversing declining vaccination trends and addressing immunity gaps, as well as extending previous gains in vaccination coverage beyond prepandemic levels, requires targeted and context-specific approaches that prioritize routine vaccination as an essential health service and improve access to vaccination across the life span.

## Footnotes

*
https://www.who.int/about/who-we-are/regional-offices

† For a given vaccine, administrative coverage is the number of doses administered in a specified target group divided by the estimated target population. Doses administered during routine immunization visits are counted, but doses administered during supplemental immunization activities (mass campaigns) usually are not. Survey-based vaccination coverage is calculated as the proportion of persons in a target age group who had received a vaccine dose. During surveys, a representative sample of households is visited, and caregivers of children in a specified target age group (e.g., 12–23 months) are interviewed. Vaccination dates are transcribed from the child’s home-based record or health facility records, and if documented evidence is unavailable, recorded based on caregiver recall.

§ For 18 countries that did not report 2021 immunization coverage data by July 7, 2021, estimated coverage for 2020 was used. https://www.who.int/teams/immunization-vaccines-and-biologicals/immunization-analysis-and-insights/global-monitoring/immunization-coverage/who-unicef-estimates-of-national-immunization-coverage

¶ The 2030 Agenda for Sustainable Development was adopted in 2015 by the 193 countries that make up the United Nations. The agenda lays out 17 Sustainable Development Goals (SDGs) and targets for dignity, peace, and prosperity for the planet and humankind, to be completed by 2030. SDG indicator SDG3.b.1 is the proportion of the target population covered by all vaccines included in their national program. https://sdgs.un.org/goals

** Gross national income (GNI) per capita is calculated using the World Bank Atlas method in U.S. dollars (USD). For all years shown, the Cook Islands and Niue are not included because GNI estimates were not available. For 2020 and 2021, data for Venezuela were also temporarily unclassified pending the release of revised national accounts statistics. https://datahelpdesk.worldbank.org/knowledgebase/articles/906519-world-bank-country-and-lending-groups

†† Gavi is a public-private global health partnership that aims to increase access to immunization in poor countries. Based on Gavi 5.0 (2021–2025), eligibility includes 57 low- and middle-income countries eligible to receive financial assistance through grants contingent on a country’s GNI per capita. Eligibility is defined as a country’s average 3-year GNI per capita in ≤1,630 USD. As GNI increases, a country moves through Gavi’s different eligibility phases until reaching the transition phase in which GNI exceeds the eligibility threshold. https://www.gavi.org

§§ Includes Angola, Armenia, Azerbaijan, Bhutan, Bolivia, Cuba, Georgia, Guyana, Honduras, Indonesia, Kiribati, Moldova, Mongolia, Sri Lanka, Timor-Leste, Ukraine, and Vietnam.

¶¶ During 2010–2019, 42 countries introduced MCV2 into their immunization schedule. In 2020, only one country introduced MCV2 into its immunization schedule. In 2021, four more countries introduced MCV2 into their immunization schedule, leaving 11 WHO member states that have yet to introduce MCV2 into their routine schedule.

*** During 2019–2021, 10 countries introduced the final dose of rotavirus vaccine into their immunization schedule.

††† Given that these countries represent approximately 6% of the global birth cohort in 2021, missing data likely had a limited impact on reported estimates.
